# Isolation and *in vitro* investigation on lactic acid bacteria for potential probiotic properties from cat feces

**DOI:** 10.3389/fvets.2024.1495745

**Published:** 2024-12-02

**Authors:** Jiali Wang, Xue Yang, Yi Peng, Jingyi Zhang, Yixin Huang, Zhijun Zhong, Haifeng Liu, Hualin Fu, Ziyao Zhou, Guangneng Peng

**Affiliations:** ^1^Key Laboratory of Animal Disease and Human Health of Sichuan Province, College of Veterinary Medicine, Sichuan Agricultural University, Chengdu, China; ^2^College of Landscape Architecture, Sichuan Agricultural University, Chengdu, China

**Keywords:** cat, lactic acid bacteria strains, probiotics, antimicrobial activity, antioxidant capacity

## Abstract

**Background:**

Probiotics, which are beneficial to the host, have been shown to benefit the health of cats. Lactic acid bacteria (LAB) are commonly used probiotics, but most strains used for cats are not derived from cats, leading to reduced efficacy and poor adaptation to cats. The objective was to identify LAB with promising probiotic potential specific to cats.

**Method:**

LABs were isolated from fecal samples of 20 healthy cats. Gram staining and the survival rate in the simulated gastrointestinal tract were used for preliminary screening. Candidate strains were identified by 16S rDNA sequencing, and further evaluated for adhesion ability, growth characteristics, antibacterial activity, antioxidant capacity, and safety.

**Results:**

24 Gram-positive isolates were identified, with 10 (F1-F10) showing robust viability in the simulated gastroenteric fluid. These 10 strains exhibited excellent adhesion to Caco-2 cells and strong auto-agglutination properties. They also possessed the capacity to antagonize and aggregate pathogens (*Staphylococcus aureus* ATCC 25923, *Salmonella Braenderup* H9812, *Escherichia coli* ATCC 25922, and *Pseudomonas aeruginosa* PAO1), Moreover, all strains demonstrated tolerance to H_2_O_2_ concentrations ranging from 0.5–2 mmol/L and the ability to scavenge 1, 1-diphenyl-2-picrylhydrazyl (DPPH) free radicals, indicating a certain level of antioxidant activity. Safety tests showed no hemolytic activity, and all but F6 were highly sensitive to antibiotics, with over 62.5% sensitivity to 16 antibiotics. Remarkably, F4 (*Lactobacillus reuteri*) and F10 (*Lactobacillus brevis*) exhibited exceptional viability in the simulated gastrointestinal tract, coupled with robust growth potential, enhanced adhesion efficiency, significant antibacterial and antioxidant properties.

**Conclusion:**

Our findings revealed that F4 (*Lactobacillus reuteri*) and F10 (*Lactobacillus brevis*) hold promising potential as probiotics. This research lays a solid scientific foundation for the selection and application of probiotics tailored specifically for cats.

## Introduction

As a companion animal, the health of cats has attracted more and more attention. Research has indicated a strong correlation between intestinal health and the overall well-being of the host (48, 1). Cats, in particular, are prone to experiencing diarrhea. This condition can arise from a variety of causes. Furthermore, they frequently struggling with intestinal issues that can pose significant risks to their life and health (2, 3). Preserving optimal intestinal health could empower them to resist intestinal damage caused by adverse external environments and pathogenic intestinal microbes (4). Consequently, it is crucial to identify effective strategies to safeguard the intestinal homeostasis of domestic cats.

Probiotics are defined as living microorganisms that are beneficial to the host when properly administered (5). Lactic acid bacteria (LAB), is the most commonly used probiotic, proven effective in maintaining cats’ intestinal health (6, 7). For instance, the research conducted by Kerek et al. highlights probiotics as a promising approach to decrease the reliance on antibiotics in companion animals (8); a multi-strain probiotic consisting of *Saccharomyces boulardii* and *Pediococcus acidilactici* promotes intestinal health in cats (9); Lappin’s study on cats infected with feline herpesvirus type 1 found that *Enterococcus faecium* SF68 preserved intestinal microbiome diversity and reduced chronic infection rates (10); Bybee showed that *Enterococcus faecium* SF68 decreased diarrhea incidence in cats (11), ande Additives verified *L. reuteri*’s benefits for cats’ intestinal health (12). Overall, LAB is beneficial for cats’ health.

Probiotics isolated from the same species of host can more effectively exhibit their probiotic characteristics (13). However, it is regrettable that most of LAB currently used in cats is not cat-specific, leading to issues such as reduced efficacy and poor adaptation to the host. To date, there is a significant gap in research on LAB derived from cats both domestically and internationally, which hinders the provision of scientific guidance for the clinical application of cat-specific probiotics. The objective of this study is to isolate LAB from the feces of healthy cats and to assess their probiotic properties. The assessment includes resistance to artificial gastric and intestinal fluids, adhesion capabilities, growth characteristics, antibacterial activity, drug resistance, antioxidant capacity, and safety. The aim is to identify LAB with promising probiotic potential specific to cats, offering a valuable reference for the application of probiotics for cats.

## Materials and methods

### Samples collection and isolation of LAB

Fecal samples from 20 healthy adult cats were collected. These cats were from three cattery houses in Chengdu that had not used antibiotics or probiotics for 2 months. The samples were collected from the rectum with sterile swabs and transferred into sterile 1.5 mL centrifuge tubes. It is then packed in ice and transported to the lab. The samples were diluted with sterile PBS and then placed in de Man, Rogosa, and Sharpe (MRS) Liquid medium (Hopebio, Qingdao, China) at 10% and incubated anaerobically at 37°C for 24 h. Then the culture medium was diluted by 10 times gradient, and 100 uL culture medium with different gradients was inoculated on MRS Solid medium. After culture for 24–48 h, single colonies were selected and re-plotted on the MRS Plate. Subsequently, these single colonies were screened and purified by continuous passage 3 times.

### Preliminary screening

#### Gram stain

The isolated and purified single colonies were enriched with MRS broth. Then, subjected to Gram staining microscopy to observe the morphology and staining of the isolated strains.

#### Tolerance for the simulated GIT condition

Utilizing the method described by Zhang, the strains identified as Gram-positive through staining were evaluated for their tolerance to simulated gastrointestinal tract (GIT) conditions (14). 0.3 g of pepsin was dissolved in 100 mL of 0.9% sterile normal saline, with pH adjusted to 3.0 using 1 M HCL to simulate gastric fluid. Simulated intestinal fluid was prepared by dissolving 0.2 g trypsin and 0.3 g cow bile salt in 100 mL of 0.9% sterile normal saline, pH adjusted to 8.0 with 1 M NaOH. Both fluids were filtered to remove bacteria. The strains were inoculated into 10 mL MRS Broth at 1% volume and cultured for 14 h. After centrifugation (25°C, 8,000 × g, 15 min), the pellets were re-suspended in sterile saline and centrifuged under the same conditions to wash 3 times. The cells were then re-suspended in 10 mL of the prepared simulated gastric juice and incubated at 37°C for 3 h anaerobically. Following this, Centrifugal collection the pellets were transferred to 10 mL of simulated intestinal fluid and cultured at 37°C for an additional 4 h (7 h) anaerobically. The number of viable colonies at 0 h, 3 h, and 7 h was determined using the plate counting method on MRS agar. The survival rate (%) was calculated as (A0/A1) × 100%. A0 denotes the number of viable bacteria at 0 h (CFU/mL) and A1 stands for the number of viable bacteria at 3 h/7 h (CFU/mL).

#### Identification of 16S rDNA

DNA extraction kit (Beijing Tiangen Biotechnology Co., LTD.) of bacteria was used to extract the DNA of the strains screened by the test of resistance to artificial gastric and intestinal fluid. The 16S rRNA gene of the strain was amplified by PCR using universal primers (F: 5’-AGAGTTTGATCCTGGCTCAG-3′; R: 5’-GGTTACCTTGTTACGACTT-3′). PCR products were then separated via 1.5% agarose gel electrophoresis, and the positive products were sent to Sangon Biotech Co. Ltd. (Shanghai, China) for sequencing. The obtained 16S rDNA sequences were compared with the Genbank database. The sequences with the highest homology were selected to construct a phylogenetic tree based on the 16S rDNA sequences by using MEGA software (version 7.0). The maximum likelihood method was employed for clustering, with 1,000 bootstrap replicates used to estimate the robustness of individual branches.

#### Assessment of the growth capacity

The growth performance of the 10 isolated strains was assessed following the methodology described in a previous study (15). 10 μL (1%) of each LAB culture was inoculated into 10 mL of fresh MRS broth and incubated at 37°C for 48 h. The absorbance at 600 nm was recorded every 2 h from 0–12 h and then every 4 h from 13–48 h. Additionally, MRS broth without inoculation served as the control group throughout the experiment.

#### Adhesion ability

According to the method reported by Anderson et al., to determine the adhesion of LAB to human Colon Colorectal adenocarcinoma cells (Caco-2) (16). Caco-2 cell line purchased from Wuhan Punos Company was cultured in DMEM medium containing 10% fetal bovine serum and 1% double antibody at 37°C Incubator with 5% carbon dioxide. Inoculate 1 mL of Caco-2 cell suspension (1.0 × 10^5^ cells/mL, Vc) into a 12-well cell culture plate and wait until about 90% of the cells are attached. Then washed 3 times with PBS and 1 mL 1.0 × 10^8^ CFU/mL (V_0_) LAB suspension (DMEM resuspension) was added to each well. After incubation at 37°C for 2 h, washed 3 times with PBS to remove unadherent bacteria. The adherent cells were digested with 0.25% pancreatic enzyme, diluted by 10 times gradient using DMEM medium, then 100 uL was applied to MRS Plate, incubated at 37°C for 24–48 h, and colony counting was performed. The adhesion rate (%) is calculated as (1 − V/V_0_) × 100.

#### Auto-aggregation and co-aggregation ability

To assess the auto-aggregation and co-aggregation abilities of the strains, the method described by Chen et al. was modified (17). The overnight cultures of LAB were adjusted to a concentration of 1 × 10^8^ CFU/mL with sterile PBS for the ao-aggregation assay. 4 mL aliquot of the bacterial suspension was used to measure the initial OD_600_ nm (A0). The suspension was then incubated at 37°C for 6 h, after which the upper suspension was sampled to measure OD_600_ nm (A). The auto-aggregation percentage (%) was calculated using the formula (1-A/A0) × 100.

For the co-aggregation assay, 2 mL of suspension was mixed with 2 mL suspension of pathogenic bacteria (*Escherichia coli* ATCC 25922, *Salmonella Braenderup* H9812, *Staphylococcus aureus* ATCC 25923, *Pseudomonas aeruginosa* PAO1) and incubated at 37°C for 6 h. The absorbance at 600 nm (A_X + Y_) was measured at the end of the incubation. The co-aggregation rate (%) was calculated using the formula [1–2 A_X + Y_ / (A_X_ + A_Y_)] × 100, where A_X_ represents the OD_600_ of the LAB suspension and A_Y_ represents the OD_600_ of the pathogenic bacteria suspension.

#### Antagonistic activity

The antibacterial properties of the strain were evaluated using the Oxford cup method as reported (18). Following an overnight culture in MRS Liquid, the supernatant was separated by centrifugation (4°C, 8000 rpm for 10 min). To remove bacteria, the supernatant was filtered through a 0.22 μm sterile filter, and adjusted to pH = 7 using 1 M NaOH to obtain the cell-free supernatant. The pathogens were inoculated in LB broth (*Escherichia coli* ATCC 25922, *Salmonella Braenderup* H9812, *Staphylococcus aureus* ATCC 25923, *Pseudomonas aeruginosa* PAO1) was cultured at 37°C for 18 h and the concentration of the bacteria was adjusted to 1 × 10^7^ CFU/mL by aseptic PBS. 100 μL of pathogen liquid was evenly spread on the LB plate. The Oxford cup was fixed on the plate and 100 μL cell-free supernatant of LAB was added, then placed in an incubator at 37°C for 24 h. Finally, the diameter of the inhibition zone was measured and recorded.

### Antioxidative ability

#### Tolerance to hydrogen peroxide (H_2_O_2_)

Following the method proposed by Li et al. with modifications (19), the 2% (v/v) overnight culture of LAB (1 × 10^8^ CFU/mL) was inoculated into MRS broth containing 0, 0.5, 1.0, 1.5, or 2.0 mmol/L H_2_O_2_. After incubation at 37°C for 8 h, the OD_600_ of the cultures was measured. The group with 0 mmol/L H_2_O_2_ served as the control. The resistance rate (%) was calculated as (OD_test_/OD_control_) × 100.

#### The ability of scavenging 1, 1-diphenyl-2-picrylhydrazyl (DPPH) free radical

Following the method reported by Lin and Chang, the DPPH free radical scavenging ability of LAB was measured (20). 2 mL of a 0.2 mmol/L DPPH solution in absolute ethanol was combined with 1 mL of either a cell-free supernatant or a bacterial suspension of the LAB strains in a centrifuge tube. The reaction was carried out in the dark at room temperature for 30 min. Then, the supernatant was collected by centrifugation (4°C, 8,000 rpm for 10 min). The OD_517_ of the supernatant was measured. Absolute ethanol was used in place of DPPH as the blank, and distilled water was used instead of the sample solution as the control. The DPPH free radical scavenging rate (%) was calculated as [1− (OD_experimental_ − OD_blank_) / ODcontrol] × 100.

### Safety assessment

#### Hemolytic activity

The LAB was inoculated on the blood plate, *Staphylococcus aureus* ATCC 25923 was used as the positive control. The results were observed after culture at 37°C for 48 h. Hemolysis was categorized into *β*-hemolysis (the complete lysis of red blood cells, resulting in a clear hemolysis ring surrounding the colony), *α*-hemolysis (partial hydrolysis of red blood cells, forming a greenish hemolysis ring around the colony), and *γ*-hemolysis (no hydrolysis of red blood cells and no hemolysis zone is observed around the colony) (21).

#### Antibiotic susceptibility

The susceptibility of LAB to antibiotics was determined according to Zhang et al. with minor modifications (22). The sensitivity of the isolated strains to 16 antibiotics was evaluated by disk diffusion test, including cefazolin (KZ, 30 μg), erythromycin (E, 15 μg), chloramphenicol (C, 30 μg), tetracycline (TE, 30 μg), ampicillin (AMP, 10 μg), clindamycin (DA, 10 μg), penicillin G (P, 10 μg), cefotaxime (CTX, 30 μg), cefuroxim (CXM, 30 μg), amikacin (AK, 30 μg), vancomycin (VA, 30 μg), oxacillin (OX, 5 μg), norfloxacin (NOR, 5 μg), fosfomycin (S, 200 μg), rifampicin (RD, 5 μg), amoxicillin (AML, 25 μg). The suspension of LAB cultured overnight was diluted to 1 × 10^8^ CFU/mL, and 100 μL was coated on the MRS plate and dried. After that, the paper containing antibiotics was placed on the surface of the MRS plate with tweezers. Then, cultured under anaerobic conditions at 37°C for 48 h. Antibiotic sensitivity was classified as resistance (R, ≤15 mm), intermediate (I, 16–20 mm), or sensitivity (S, ≥21 mm) by measuring the diameter of the inhibition zone (mm), according to the parameters of the Institute of Clinical and Laboratory Standards (13).

### Statistical analysis

The results are expressed as mean ± standard deviation (SD) and were analyzed using the SPSS 19.0 statistical package (SPSS Inc., Chicago, USA). Significance was determined at *p* < 0.05 for statistically significant differences and at *p* < 0.01 for extremely significant differences. The figures were created using GraphPad Prism 9.5 (GraphPad Software Inc., La Jolla, USA).

## Results

### Preliminary screening

**Figure 1:**
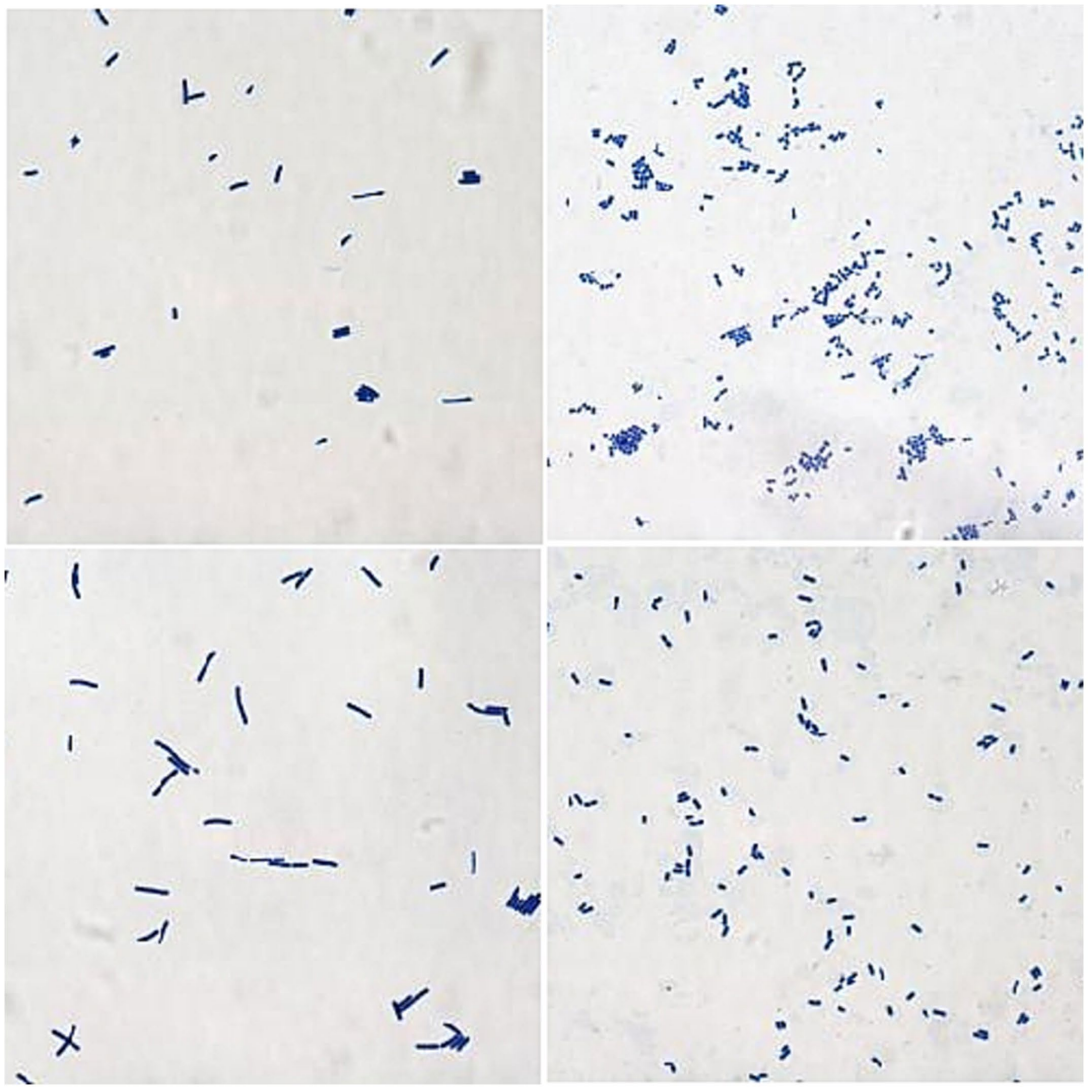
The results of Gram staining for parts of the isolates (1000×).

The isolated and purified strains were examined by microscopy after Gram staining. The staining results of 24 isolates were Gram-positive, which was consistent with the staining characteristics of LAB strains. Colony morphologies include spheres, short rods, and long rods (Figure 1).

**Figure 2:**
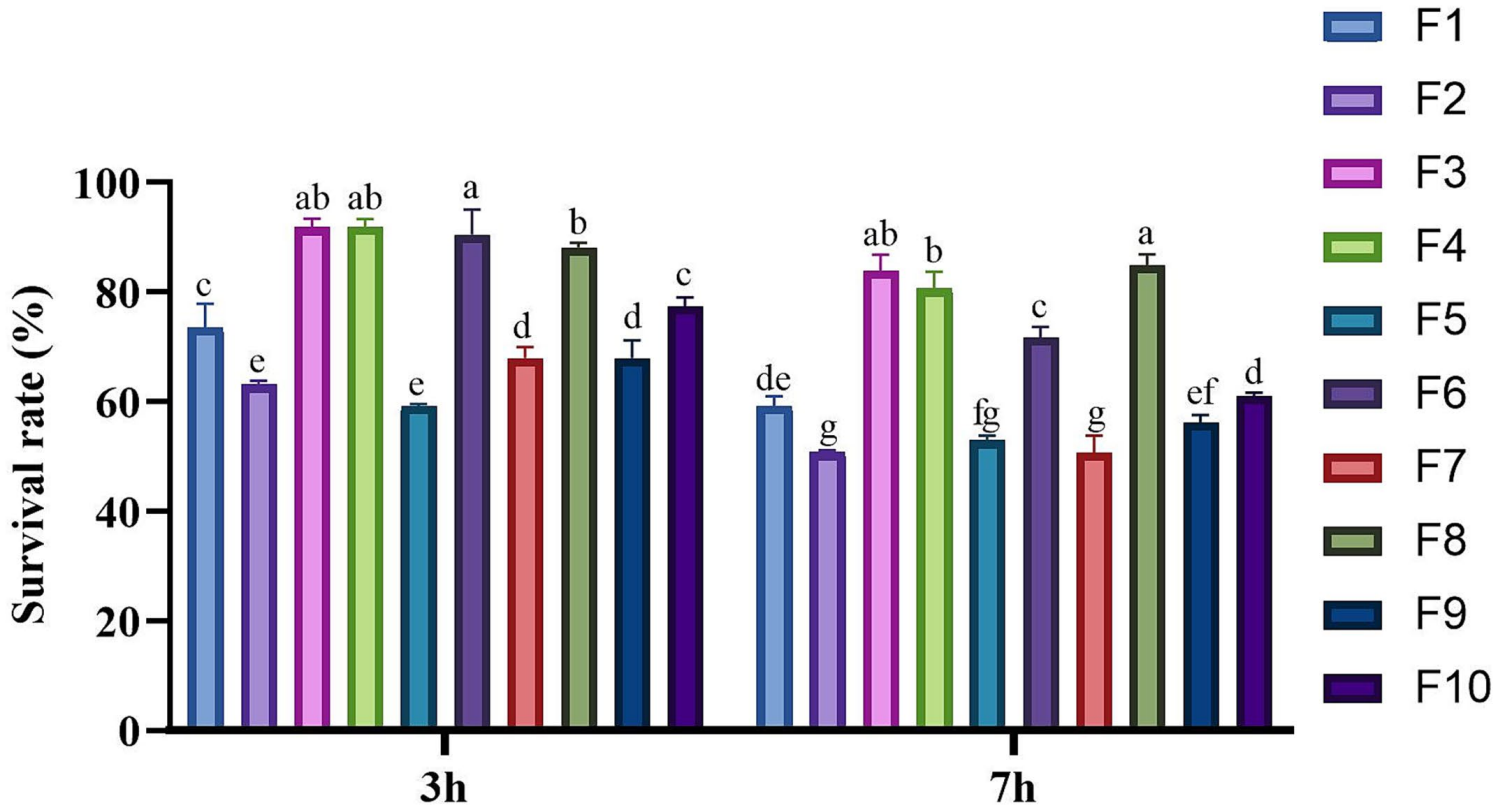
Survival rate of the isolates in the simulated GIT condition. Different letters marked above each column represent significant differences (Waller-Duncan, *p* < 0.05).

The resilience of these 24 isolates to conditions simulating the GIT was subsequently assessed. It was found that only 10 isolates demonstrated remarkable tolerance, with a survival rate exceeding 50% under simulated GIT conditions (Figure 2). The survival rates of these 10 isolated strains varied between 59.17–84.85%. Notably, the three strains with the highest survival rates were F3 (83.91%), F4 (80.59%), and F8 (84.85%), each exhibiting survival rates exceeding 80%, which suggests a robust tolerance in the gastrointestinal tract GIT conditions. In contrast, the remaining 14 strains exhibited poor tolerance suggesting that they may not adequately survive after oral administration in a living organism. Consequently, only the 10 isolates with high survival rates in the GIT simulation were selected for further evaluation in subsequent experiments. Based on their morphological characteristics, these 10 strains were named F1 - F10.

### Species identification

**Figure 3:**
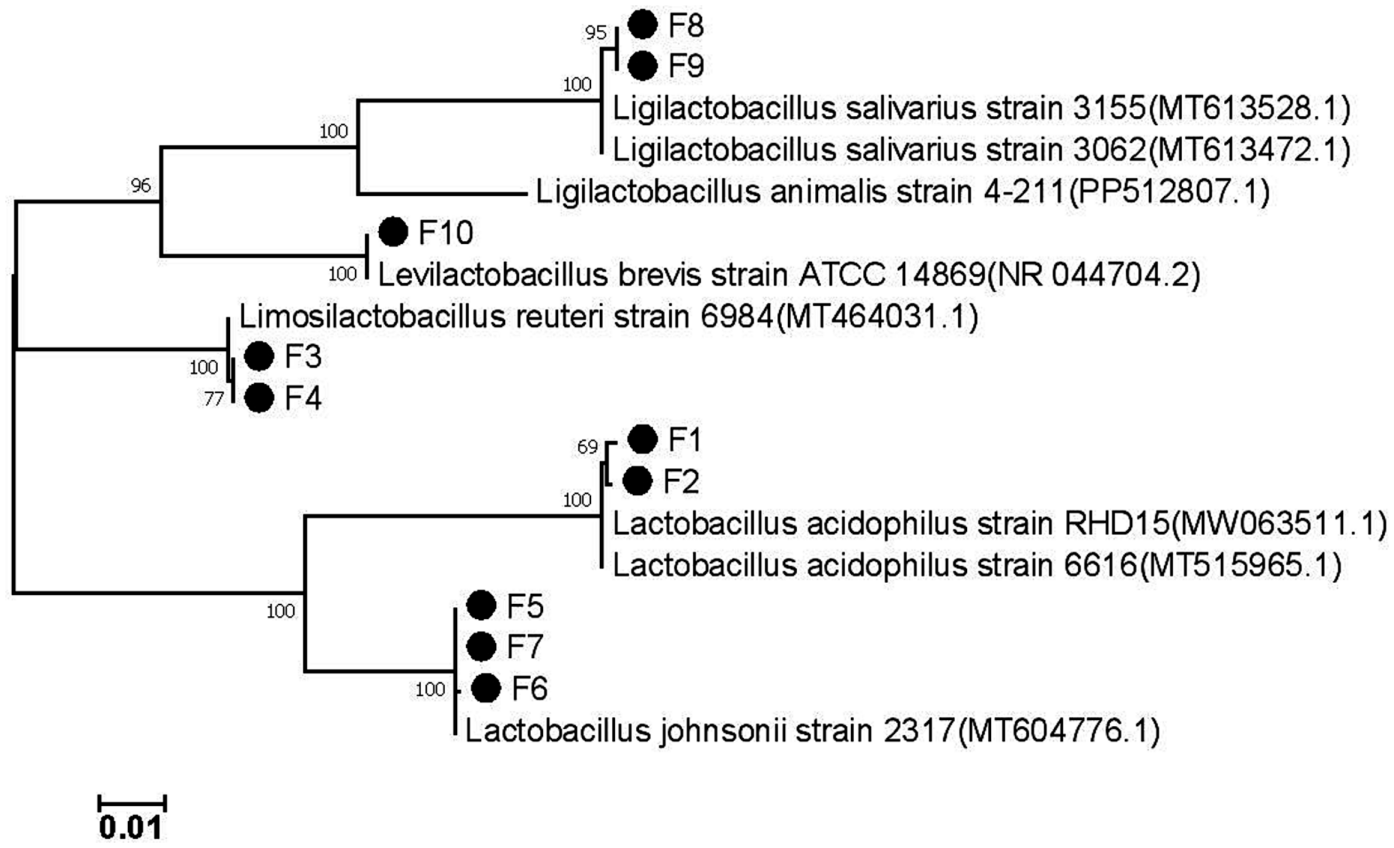
The phylogenetic tree of the isolates based on 16S rDNA genes.

The 16S rRNA gene sequence of the isolated strain was successfully amplified using PCR, and the sequence has been deposited in GenBank under the accession numbers PP989619 to PP989628. The most closely related reference sequence in GenBank was retrieved, and a phylogenetic tree based on the 16S gene was constructed using MEGA7.0 software (Figure 3). The analysis revealed that the 10 isolated strains comprised *Lactobacillus acidophilus* (F1, F2), *Lactobacillus reuteri* (F3, F4), *Lactobacillus johnsonii* (F5, F6, F7), *Lactobacillus salivarius* (F8, F9), and *Lactobacillus brevis* (F10). This classification aligns with the previous morphological clustering, indicating that strains with similar appearances are indeed the same species of LAB.

### Growth ability

**Figure 4:**
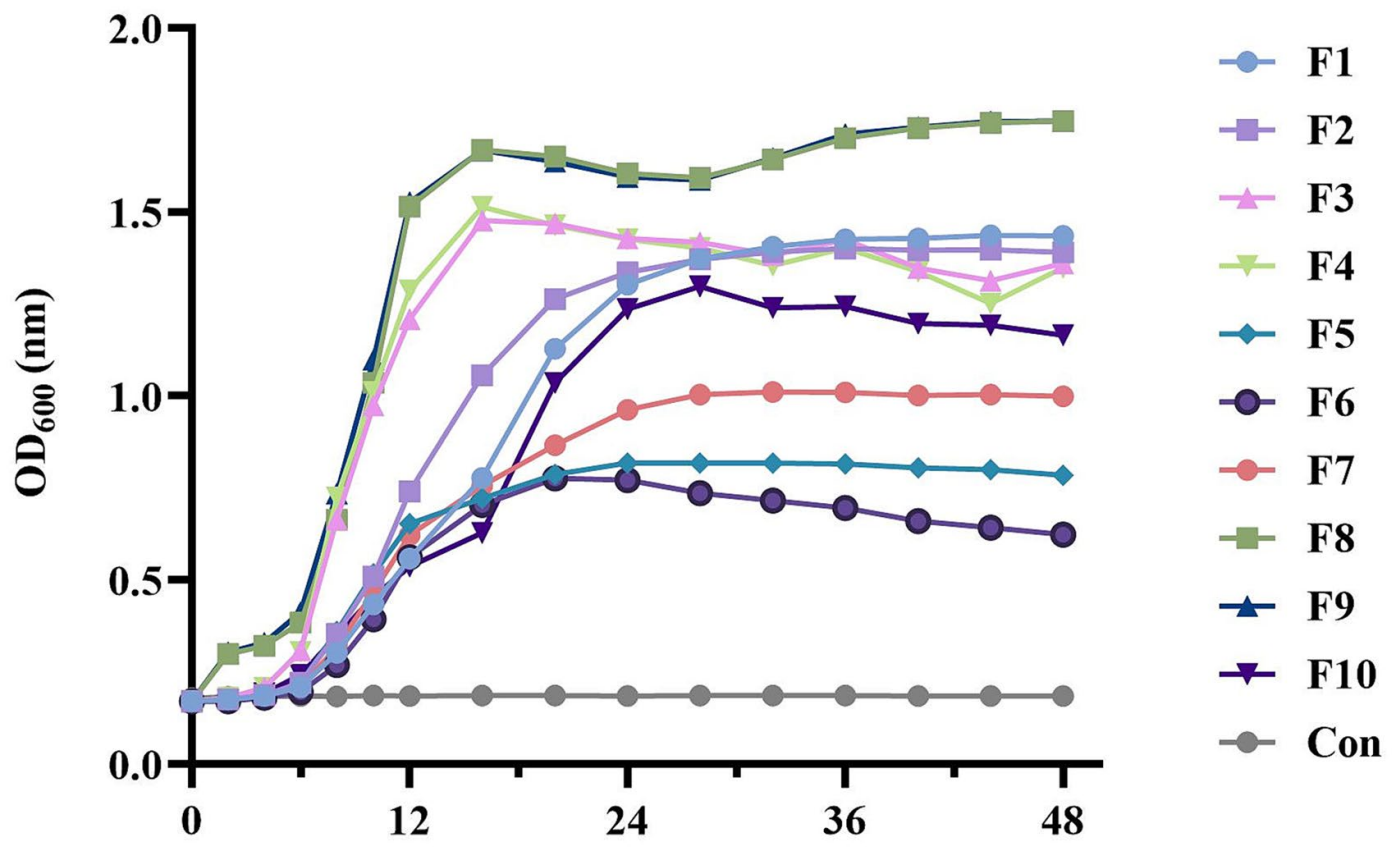
Growth curves of the LAB isolates. All the results are represented as mean ± SD.

Analysis of the growth curves for the isolated strains in Figure 4 revealed distinct growth patterns. Notably, strains of the same species exhibited consistent growth characteristics. *Lactobacillus reuteri* (F3, F4) and *Lactobacillus salivarius* (F8, F9) exhibited rapid proliferation, reaching the stable growth phase around 12 h, especially for F8 and F9, the OD_600_ of these two strains exceeded 1.5, reaching 1.51 (F8) and 1.53 (F9) respectively, indicating strong growth capabilities; *Lactobacillus acidophilus* (F1, F2) and *Lactobacillus brevis* (F10) displayed a slower growth rate than the above four strains, OD_600_ ranging from 1–1.5, was lower than that of the four strains above. However, *Lactobacillus johnsonii* (F5, F6, F7) showed the poorest growth performance, entering the stable phase around 24 h. Significantly, the OD_600_ of these three strains were all lower than 1, which showed the lowest bacterial concentration compared with other strains.

### Adhesion to Caco-2 cell line

**Figure 5:**
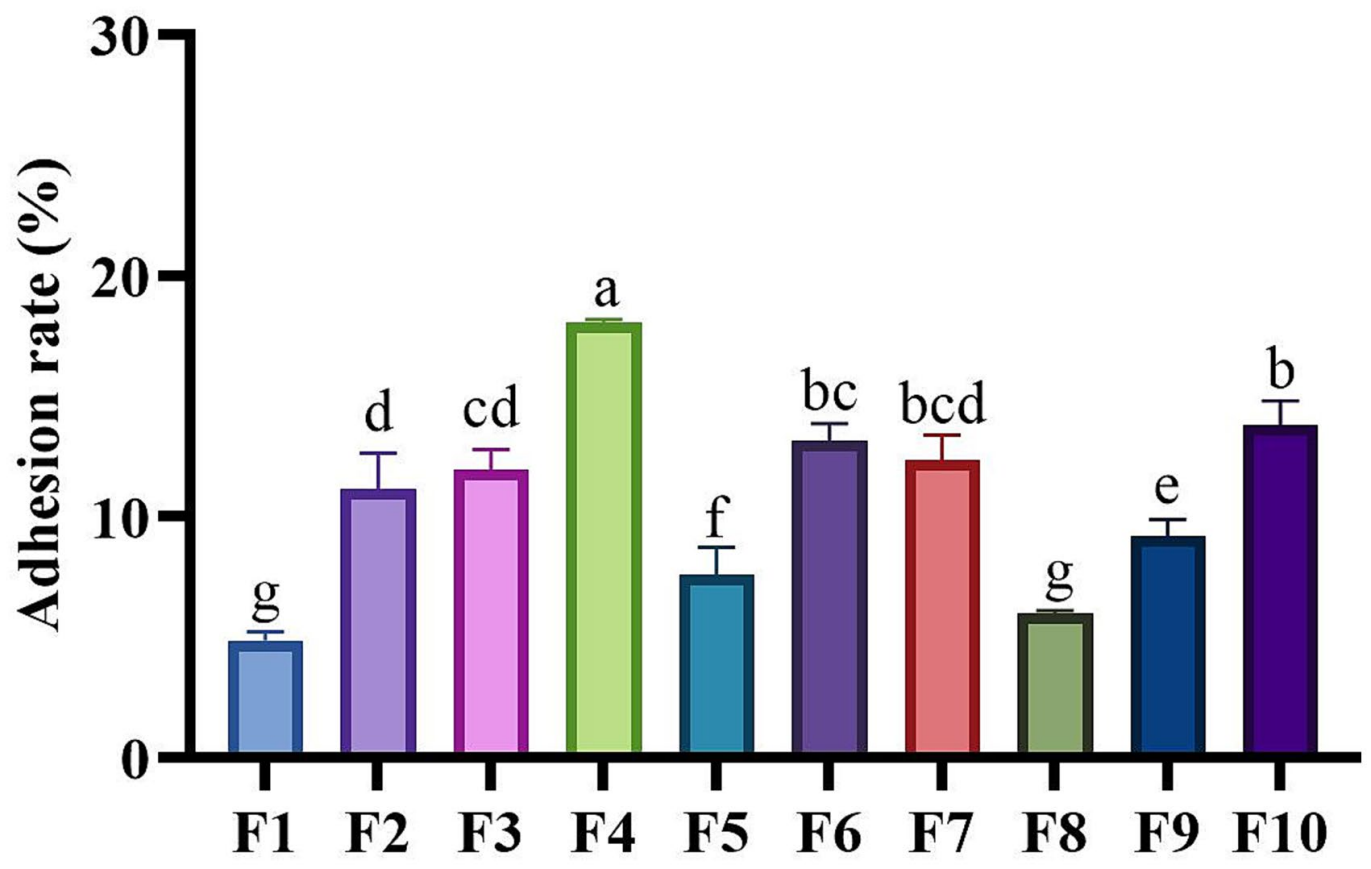
Adhesion capabilities of LAB isolates to Caco-2 cell line. Different letters marked above each column represent significant differences (Waller-Duncan, *p* < 0.05).

Figure 5 illustrates the adhesion rates of the isolated strains to Caco-2 cells. The adhesion rates of the strains in this study varied between 4.87% and 18.05%. With the exception of four strains: F1 (4.87%), F5 (7.59%), F8 (5.99%) and F9 (9.91%), all other strains exhibited adhesion rates exceeding 10%. Notably, F4 demonstrated the highest adhesion capacity, reaching 18.5%, followed by F10 (13.81%) and F6 (13.16%). In contrast, F1 had the lowest adhesion rate (4.87%).

### Auto-aggregation and Co-aggregation ability

Based on the results of auto-aggregation and co-aggregation shown in Table 1, the isolated strains exhibited varying aggregation abilities. Among the 10 LAB strains isolated in this study, the top three with the highest auto-aggregation ability were F2 (88.44%), F4 (86.79%), and F6 (81.71%), each with an auto-aggregation rate exceeding 80%. The strains demonstrating the high co-aggregation potential with *Staphylococcus aureus* ATCC 25923 were identified as F2 (87.44%), F5 (78.52%), and F3 (77.69%). In the case of *Salmonella Braenderup* H9812, the effective co-aggregating strains were F6 (69.64%), F2 (68.77%) and F9 (68.76%). For *Escherichia coli* ATCC 25922, the strains that exhibited the top three co-aggregation capacity were F6 (68.01%), F1 (67.23), and F2 (66.69%). Lastly, in the context of *Pseudomonas aeruginosa PAO1*, the strains with robust co-aggregation ability were found to be F6 (70.89%), F2 (70.12%), and F9 (69.16%).

**Table 1:** Auto-aggregation and Co-aggregation ability of the isolated 10 LAB.

Strains	Auto-aggregation rate (%)	Co-aggregative rate (%)			
		*Staphylococcus aureus* ATCC 25923	*Salmonella Braenderup* H9812	*Escherichia coli* ATCC 25922	*Pseudomonas aeruginosa* PAO1
F1	36.66 ± 1.79i	77.67 ± 2.33b	62.19 ± 0.46bc	67.23 ± 0.61ab	63.36 ± 0.87bc
F2	88.44 ± 1.04a	87.44 ± 0.5a	68.77 ± 1.34a	66.69 ± 1.46abc	70.12 ± 1.01a
F3	52.38 ± 0.22f	77.69 ± 1.14b	60.13 ± 2.47c	58.57 ± 1.66d	62.67 ± 1.95bc
F4	86.79 ± 1.36b	65.21 ± 0.52d	65.19 ± 0.56b	63.79 ± 0.63c	66.51 ± 0.66abc
F5	43.4 ± 0.58 h	78.52 ± 1.6b	55.04 ± 2.01d	57.44 ± 0.43d	67.03 ± 10.68abc
F6	81.71 ± 0.19c	71.33 ± 1.04c	69.64 ± 1.57a	68.01 ± 2.03a	70.89 ± 1.12a
F7	45 ± 0.03 g	47.64 ± 3.84e	53.42 ± 3.8d	25.42 ± 4.25f	48.53 ± 0.35d
F8	57.02 ± 0.32e	67.02 ± 0.72d	65.08 ± 0.48b	64.32 ± 0.43bc	68.51 ± 0.33ab
F9	56.65 ± 0.6e	70.02 ± 1.75c	68.76 ± 0.33a	64.56 ± 0.81bc	69.16 ± 1.67ab
F10	76.94 ± 0.63d	64.68 ± 0.85d	59.21 ± 1.81c	52.67 ± 0.88e	60.68 ± 0.54c

Results of independent experiments (*n* = 3) are presented using mean ± SD; Superscripts with different letters represent significantly different values in the same column (Waller-Duncan, *p* < 0.05).

### Antimicrobial ability

Table 2 illustrates the inhibitory effects of 10 LAB isolates against four prevalent intestinal pathogens. The efficacy of these strains in inhibiting different pathogens varies significantly. Research has demonstrated that the antibacterial capacity of LAB can be classified into four levels based on the diameter of the inhibition zone: I (8 mm < zone diameters ≤12 mm), II (12 mm < zone diameters ≤16 mm), III (16 mm < zone diameters ≤20 mm), and IV (20 mm < zone diameters) (23). Compared the diameters of the inhibition zones of all strains, and the results showed that F9 (19.21 mm), F4 (19.14 mm), and F8 (17.88 mm) exhibit potent inhibitory effects against *Escherichia coli* ATCC 25922; F6 (30.67 mm), F1 (27.4 mm), and F2 (17.46 mm) demonstrate strong inhibition against *Staphylococcus aureus* ATCC 25923; F10 (23.32 mm), F3 (22.04 mm), and F2 (17.41 mm) are particularly effective against *Salmonella Braenderup* H9812; F10 (28.59 mm), F5 (26.36 mm), and F1 (26.13 mm) show significant inhibitory effects on *Pseudomonas aeruginosa* PAO1. Moreover, except for F7 (17.97 mm) and F8 (19.13 mm), whose inhibition diameters against *Pseudomonas aeruginosa* PAO1 were at the III level (16 mm < zone diameters ≤20 mm), the inhibition diameters of the other isolated strains were all at the IV level (20 mm < zone diameters).

**Table 2:** Antimicrobial ability of the isolated 10 LAB.

Strains	Antagonistic activity (mm)			
	*Staphylococcus aureus* ATCC 25923	*Salmonella Braenderup* H9812	*Escherichia coli* ATCC 25922	*Pseudomonas aeruginosa* PAO1
F1	27.4 ± 1.23b	14.4 ± 0.3c	12.37 ± 0.14c	26.13 ± 1.45ab
F2	17.46 ± 1.81c	17.41 ± 2.07b	13.36 ± 0.1c	20.76 ± 1.92c
F3	15.86 ± 0.45c	22.04 ± 2.56a	17.23 ± 0.58b	24.72 ± 1.04b
F4	12.72 ± 0.39d	11.49 ± 1.79c	19.14 ± 0.55a	23.9 ± 0.9b
F5	13.6 ± 0.88d	13.59 ± 0.92c	8.99 ± 0.84d	26.36 ± 0.36ab
F6	30.67 ± 0.63a	14.38 ± 1.68c	12.94 ± 0.81c	24.81 ± 1.85b
F7	13.69 ± 0.87d	11.64 ± 1.82c	12.43 ± 0.91c	17.97 ± 0.95d
F8	16.69 ± 1.28c	13.78 ± 0.87c	17.88 ± 0.11b	19.13 ± 1.74 cd
F9	16.03 ± 0.81c	12.41 ± 0.97c	19.21 ± 1.19a	25.51 ± 2.69b
F10	12.53 ± 0.31d	23.32 ± 0.18a	16.73 ± 0.55b	28.59 ± 0.87a

Results of independent experiments (*n* = 3) are presented using mean ± SD; Superscripts with different letters represent significantly different values in the same column (Waller-Duncan, *p* < 0.05).

### Analysis of antioxidant activity

**Figure 6:**
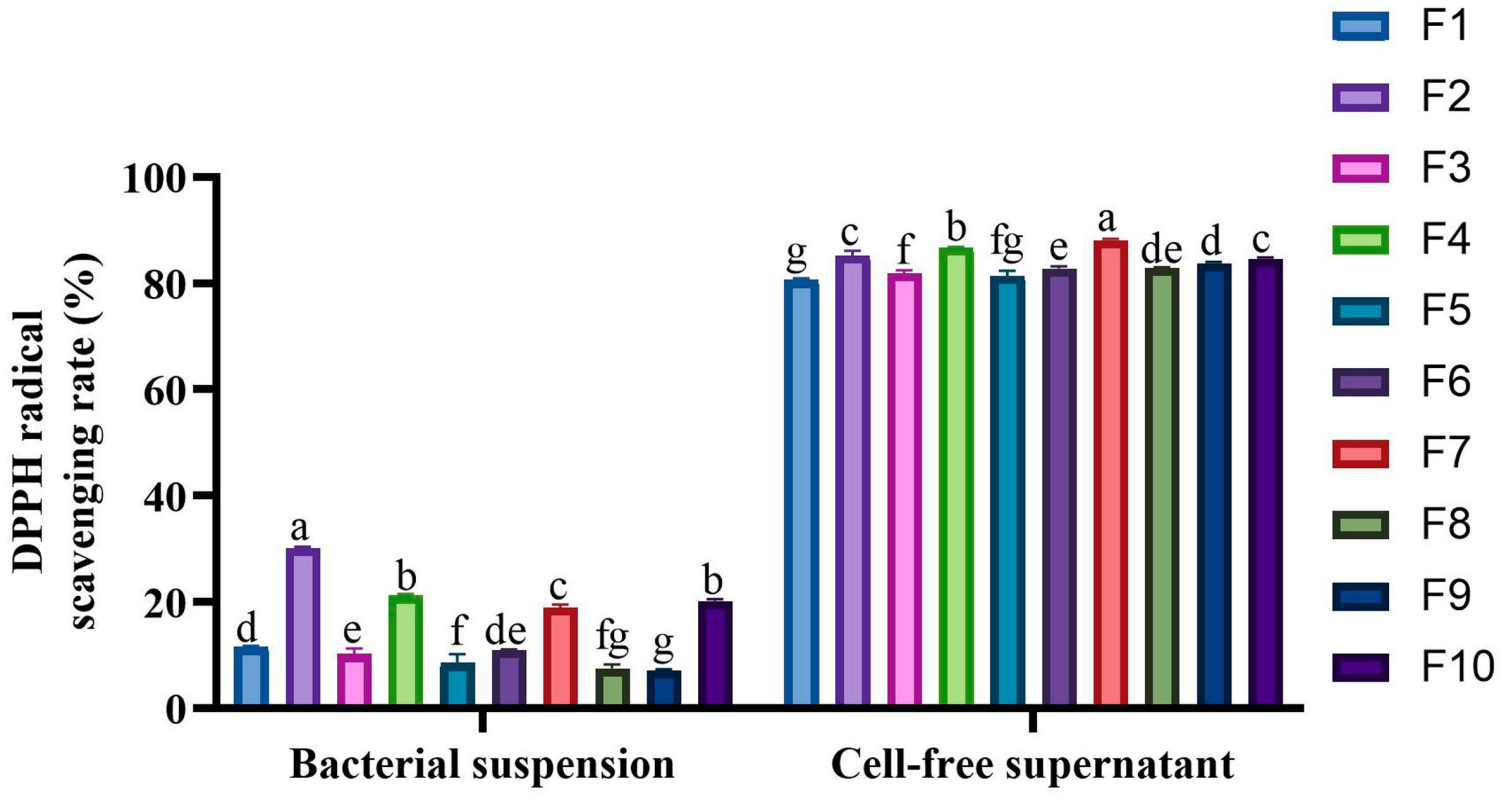
DPPH radical scavenging activity of the 10 LAB strains. Different letters marked above each column represent significant differences (Waller-Duncan, *p* < 0.05).

Tables 3 and Figure 6 present the findings on the isolated strains’ capacity to withstand H_2_O_2_ and neutralize DPPH free radicals, respectively. The data reveal that all 10 isolates exhibited tolerance to varying concentrations of H_2_O_2_, with their resilience diminishing as the H_2_O_2_ concentration intensified. The most tolerant strains, in descending order, were identified as F10 (6.44%), F4 (4.01%), and F6 (3.62%) in the highest concentration (2.0 mmol/L H_2_O_2_). In the scavenging of DPPH free radicals, the cell-free supernatant from the isolated strains demonstrated a superior clearance rate compared to the bacterial suspensions. The cell-free supernatant achieved clearance rates ranging from 80.62–88.12%, whereas the bacterial suspensions only managed rates between 7.18–30.21%. Notably, the cell-free supernatant of F7 (88.12%), F4 (86.71%), and F2 (85.24%) emerged as the top 3 performers in this regard.

**Table 3:** Survival rate of the 10 LAB isolates in H_2_O_2_.

Strain	Survival rate (%)			
	0.5 mmol/L H_2_O_2_	1.0 mmol/L H_2_O_2_	1.5 mmol/L H_2_O_2_	2.0 mmol/L H_2_O_2_
F1	92.43 ± 0.36bc	4.93 ± 0.41 g	3.78 ± 0.01e	3.54 ± 0.09 cd
F2	99.67 ± 3.19a	57.56 ± 3.81b	4.35 ± 0.05d	3.31 ± 0.44cde
F3	98.69 ± 1.58a	4.32 ± 0.02 g	3.82 ± 0.07e	3.39 ± 0.15 cd
F4	89.6 ± 3.47c	36.74 ± 2d	4.74 ± 0.08c	4.01 ± 0.02b
F5	65.71 ± 1.37e	11.09 ± 1.1f	2.83 ± 0.14f	2.09 ± 0.17f
F6	80.41 ± 2.07d	27.91 ± 0.42e	8.1 ± 0.39b	3.62 ± 0.16c
F7	84.37 ± 0.91d	43.47 ± 0.53c	3.81 ± 0.07e	3 ± 0.19e
F8	96.29 ± 0.4ab	3.77 ± 0.07 g	3.62 ± 0.05e	1.64 ± 0.26 g
F9	96.71 ± 3.11ab	4.15 ± 0.24 g	3.58 ± 0.05e	3.21 ± 0.01de
F10	92.88 ± 4.16bc	81.01 ± 0.53a	39.74 ± 0.53a	6.44 ± 0.04a

Results of independent experiments (*n* = 3) are presented using mean ± SD; Superscripts with different letters represent significantly different values in the same column (Waller-Duncan, *p* < 0.05).

### Safety analysis

The hemolytic test results indicated that none of the 10 LAB isolates from this study exhibited hemolytic activity. Furthermore, the antimicrobial resistance assessment of these isolates against 18 antibiotics revealed that all strains were susceptible to rifampicin (RD) but demonstrated resistance to norfloxacin (NOR). Notably, F6 displayed the highest level of drug resistance, with a sensitivity rate of only 23%, being susceptible to chloramphenicol (C), amikacin (AK), lecithin (S), and rifampicin (RD). In contrast, F2 had the highest sensitivity rate at 87.5%; followed by F4, F5, and F7, with their sensitivity rates all being 81.25% (Table 4).

**Table 4:** Antibiotic susceptibility of the 10 LAB strains.

Strain	Antibiotic susceptibility																Sensitive rate % (S + I, %)
	KZ	E	C	TE	AMP	DA	P	CTX	CXM	AK	VA	OX	NOR	S	RD	AML	
F1	S	S	S	R	S	S	S	S	S	R	R	R	R	R	S	S	62.5
F2	S	S	S	I	S	S	S	S	S	S	R	S	R	I	S	S	87.25
F3	S	R	S	S	S	S	S	S	S	I	R	R	R	R	S	S	68.75
F4	S	S	S	S	S	S	S	S	S	S	R	R	R	S	S	S	81.25
F5	S	S	S	S	S	S	S	S	S	R	S	R	R	I	S	S	81.25
F6	R	R	S	R	R	R	R	R	R	S	R	R	R	S	S	R	23
F7	S	S	S	S	S	I	S	S	S	R	S	I	R	R	S	S	81.25
F8	S	S	S	R	S	S	S	S	S	R	R	R	R	R	S	S	76.92
F9	I	S	S	R	S	S	S	S	S	R	R	R	R	R	S	S	62.5
F10	S	S	S	S	S	S	S	S	S	R	R	R	R	R	S	S	68.75

KZ, cefazolin; E, erythromycin; C, chloramphenicol; TE, tetracycline; AMP, ampicillin; DA, clindamycin; P, penicillin G; CTX, cefotaxime; CXM, cefuroxim; AK, amikacin; VA, vancomycin; OX, oxacillin; NOR, norfloxacin; S, fosfomycin; RD, rifampicin, and AML, amoxicillin. R, Resistance; S, Sensitive; and I, Intermediate.

## Discussion

Probiotics offer multiple benefits, including improved intestinal health, enhanced antioxidant and immune functions, and pathogen inhibition, reducing antibiotic reliance and addressing resistance (24). Probiotics must be well-adapted and safe for the host (25), hence isolating them from similar hosts improves gastrointestinal tract adaptation and efficacy. Studies show probiotic benefits in cats, but most strains are not cat-derived (26, 27), limiting diversity and potential efficacy. This study isolates LAB from healthy cats to provide more appropriate and effective probiotics for cats’ health.

LAB are Gram-positive, appearing purple when subjected to Gram staining (28). Based on this characteristic, we preliminary screened the strains isolated and purified from MRS plates, ultimately identifying 24 Gram-positive strains as potential LAB candidates. For probiotics to be effective, they must survive the harsh conditions of the gastric and intestinal fluids after oral administration (29). Therefore, we initially assessed the gastrointestinal fluid tolerance of all suspected LAB isolates by simulating gastrointestinal conditions, resulting in the selection of 10 strains with strong tolerance. These strains are likely to have a high survival rate in the host after oral administration, ensuring effective utilization. Species identification through the 16S rDNA gene analysis confirmed that all 10 isolated strains were indeed LAB, consistent with the Gram staining results. These strains were classified into five species: *Lactobacillus acidophilus* (F1, F2), *Lactobacillus reuteri* (F3, F4), *Lactobacillus johnsonii* (F5, F6, F7), *Lactobacillus salivarius* (F8, F9), and *Lactobacillus brevis* (F10). This study has expanded the diversity of LAB isolated in cats.

As we know, probiotics are typically introduced into the host body via oral administration as living organisms to elicit their beneficial effects. Only those strains capable of adhering to the intestinal lining upon ingestion can successfully colonize and subsequently unleash their probiotic properties. Strains that fail to comply effectively are expelled from the host’s body along with the intestinal contents, leading to diminished strain efficacy, reduced probiotic impact, and potentially rendering the probiotics ineffective in the host (30). In 2018, Deng et al. reported that among strains of the same type, those with better adhesion rates had better probiotic effects, confirming that the adhesion rate of probiotics is closely related to their probiotic properties (31). In addition, probiotics can also compete with intestinal pathogens for colonization through strong adhesion, thereby exerting an antibacterial effect (32). Therefore, the ability to adhere and proliferate effectively is vital for cats’ probiotics to exert their beneficial effects and ensure high rates of utilization (33). The cell line Caco-2 is widely used as a laboratory model and is considered an important screening method for evaluating the adhesion ability of probiotics (34). The results showed that the adhesion rates of the 10 isolated strains ranged from 4.87–18.5%, consistent with the findings reported by Vidhyasagar et al., who observed adhesion rates between 9 and 17% in their isolated LAB strains from idly batter (35); Additionally, the LAB isolated from dog feces by Liu et al. exhibited a maximum adhesion rate of merely 3.62% (36), highlighting the robust adhesion capabilities of the strains in the current study. This comparison suggests that there are significant differences in the adhesion rates of LAB sourced from various origins. Of course, these disparities may be attributed to the inherent specificity of the bacterial strains themselves. Growth evaluation showed that different species of LAB had different growth abilities. While strains within the same species exhibited similar growth characteristics. Among the isolates, *Lactobacillus johnsonii* strains (F5, F6, and F7) demonstrated the most inferior growth performance, which suggests that LABs of the same species have comparable growth performance. In contrast, a study by Zhang et al. involved the isolation of five strains of *Lactobacillus plantarum* from cow’s milk, revealing distinctly different growth curves among these strains (22). When considered alongside the results of the present study, it becomes evident that the growth characteristics of LAB are not solely strain-specific but are also influenced by a range of other factors. These factors may include genetic variations within the strain’s genome, the composition of the culture environment, and the availability of nutrients, among others. Further research is necessary to fully understand the specific factors contributing to the differential growth characteristics observed in LAB strains. It is recommended that the selection of probiotics be tailored to the specific application scenarios and conditions to ensure optimal growth performance of the chosen strains.

The auto-aggregation capability of probiotics aids in the adherence of bacterial cells to the intestinal epithelial wall, while their co-aggregation ability helps prevent pathogen colonization in the intestine (37). The auto-aggregation rates of the strains isolated in this study ranged from 36.66–88.44%, with their co-aggregation potential reaching 25.82–87.44%. Significant differences were noted among the strains, yet overall, they outperformed the LAB strains isolated from panda feces by Wang et al., which had self-aggregation rates of 11.83–41.61% and co-aggregation rates of 7.89–30.15% (38). This demonstrates the robust self-aggregation and co-aggregation abilities of the LAB isolated here. Research indicates that LAB hold promise as an alternative to antibiotics in combating pathogen infections. This study evaluated the antibacterial capacity of the isolated strains against four common intestinal pathogens (*Escherichia coli* ATCC 25922, *Salmonella Braenderup* H9812, *Staphylococcus aureus* ATCC 25923, *Pseudomonas aeruginosa* PAO1). The results revealed that all strains exhibited excellent antibacterial properties, the majority of their antibacterial diameters fall into III level (16 mm < zone diameters≤20 mm) or IV level (20 mm < zone diameters), which is considerably superior to the antibacterial capabilities of LAB isolated in other reports (23, 39). In their studies, the most antibacterial diameters were classified as level II (12 mm < zone diameters ≤16 mm). Moreover, Among the four pathogens, the strain isolated in this study had the best inhibitory effect on *Pseudomonas aeruginosa* PAO1. This suggests that cat-derived LAB may produce substances that can effectively antagonize the *Pseudomonas aeruginosa* PAO1, which may be specific bacteriocins or antibacterial enzymes (32, 40). However, the specific antibacterial substances and mechanisms need further study. In conjunction with the inhibition results against the four pathogens, F1, F4, and F10 demonstrated strong comprehensive antibacterial abilities. This result provides a reference for the selection of clinical antibacterial probiotics for cats, which is beneficial to reduce the use of antibiotics and the problem of drug resistance.

An imbalance between pro-oxidants and antioxidants in the body leads to an overproduction of reactive oxygen species (ROS), causing oxidative stress. This can lead to inflammation, organ damage, and other diseases (41). Research indicates that certain LAB strains possess significant antioxidant potential, which can reduce the risk of oxidative damage to host cells and lower the incidence of chronic diseases, qualifying them as effective natural antioxidants (42). The evaluation results of the antioxidant capacity of the isolated strains in this study showed that all strains showed tolerance to H_2_O_2_ concentrations of 0.5-2 mmol/L, among which F10 showed the highest tolerance, with the survival rate of 6.44%. In contrast, in Wang’s report, only 2 of the 5 LAB isolated from panda feces could tolerate H_2_O_2_ at 2 mmol/L (38). Moreover, our study found that the supernatant of the isolated strains had a significantly higher DPPH free radical clearance rate (80.62–88.12%) than the bacterial suspension (7.17–30.21%), which is aligns with Zhou et al.’s report (43). This could be attributed to the fact that the supernatant of LAB is abundant in antioxidant metabolites, such as Exopolysaccharides (EPS) (44). The metabolites are secreted by the strains as they grow. To guarantee that the strains demonstrate superior antioxidant properties, probiotic strains must possess outstanding growth and proliferation abilities within the body. This ensures the sustained and consistent production of beneficial metabolites, thereby enhancing the probiotics’ exceptional antioxidant capabilities. However, the precise substances responsible for the antioxidant effects in our isolated strains require further experimental confirmation. Notably, F7 had the strongest free radical scavenging ability, reaching 88.12%, which, although lower than the 95.98% clearance rate of *Lactobacillus plantarum* GXL94 isolated from capsicum by Zhou (43), but significantly higher than the 57–66% rate of two LAB strains isolated from dog feces by Zhao et al. (13). These differences may stem from variations in strain origin or complex antioxidant mechanisms (45). In conclusion, the strains isolated in this study demonstrated robust antioxidant capacity, highlighting their potential in cats as natural antioxidants and their suitability for reducing oxidative stress.

The hemolysis test and antibiotic resistance profiling are crucial for assessing the safety of probiotics. Fortunately, none of the 10 strains isolated in this study exhibited hemolytic activity. In this study, the least sensitive isolate, F6, was only susceptible to 4 of the 16 tested antibiotics, indicating a relatively lower safety profile compared to the other strains, which all exhibited higher sensitivities, exceeding 62.4%. Notably, all strains showed resistance to norfloxacin, which is hypothesized to be an inherent resistance trait of these strains (46), unrelated to the presence of resistance genes. Additionally, all 10 LABs isolated here belonged to the Lactobacillus genus, a group within LAB that is generally recognized as safe (GRAS) (47). In summary, all the isolates in this study can be considered safe.

## Conclusion

In this study, 24 strains of LAB were isolated from the feces of 20 healthy cats, and 10 strains with high survival rates in the simulated GIT environment were selected to further study. Comprehensive analysis of these metrics indicated that F4 (*Lactobacillus reuteri*) and F10 (*Lactobacillus brevis*) demonstrated excellent performance and hold significant potential as probiotics. This study provides a scientific basis for the selection and application of cat-derive probiotics. However, it is important to note that these *in vitro* evaluations are not sufficient to confirm the prebiotic properties of these LAB strains *in vivo*, and further research is needed to determine whether their effects in cats align with the *in vitro* findings. Additionally, there are hundreds of LAB species, this study only isolates 5 species of *Lactobacillus*. Consequently, the findings are subject to certain limitations. Future research can enhance the scope by increasing the sample size to isolate a wider array of cat-derived LAB, thereby expanding our understanding of their diversity and probiotic potential.

## Data Availability

The datasets presented in this study can be found in online repositories. The names of the repository/repositories and accession number(s) can be found in the article/supplementary material.
